# Collaborative research networks as a strategy to synthesize knowledge of Amazonian biodiversity

**DOI:** 10.1098/rspb.2025.2069

**Published:** 2025-11-26

**Authors:** Bethânia Oliveira Resende, Leandro Juen, Juliana Schietti, Fabricio Beggiato Baccaro, Eduardo Krempser, James Moura Jr., Lucas Colares, Raquel L. Carvalho, Oliver L. Phillips, Gahelyka Souza, Thaisa Sala Michelan, André Ricardo Ghidini, André Demori, Bruna Letícia Barreto Façanha, Clarissa Rosa, Eduardo Venticinque, Francieli Bomfim, Marcelo Raseira, Shirley Famelli, Adriane Esquivel-Muelbert, Ana Claudia Kasseboehmer, Ana Luísa Fares, Anthony Santana Ferreira, Camila Cherem Ribas, Carolina Castilho, Cássio Alencar Nunes, Dárlison F. C. de Andrade, Divino Silvério, Erlane José Rodrigues Cunha, Ettore Antunes, Eva Rodrigues, Everton Silva, Fernanda Alves-Martins, Fernando Augusto Schmidt, Fernando Elias, Fernando Geraldo de Carvalho, Fernando Z. Vaz-de-Mello, Gabriel Cruz, Giovanni Palheta, Grazielle Teodoro, Hans ter Steege, James Albert, Jeanne Nascimento, Joás Brito, José Júlio Toledo, José Max B. Oliveira-Junior, Josinete Monteles, Julia Arieira, Juliana Stropp, Karina Dias-Silva, Leandro Castello, Leandro L. Giacomin, Leandro Schlemmer Brasil, Lenize Calvão, Letícia Vieira, Lis Stegmann, Luciano Montag, Marcos Pérsio Dantas Santos, Marcos Silveira, Mayerly Alexandra Guerrero-Moreno, Nathália Nascimento, Neusa Hamada, Onildo Marini-Filho, Pablo Melo, Palmira Ferreira, Paulo de Marco Júnior, Rafaella Maciel, Raimunda Pinheiro, Raphael Ligeiro, Renato Lima, Renato Martins, Ricardo Koroiva, Rogerio R. Silva, Sabina Ribeiro, Thiago Bernardi Vieira, Victor Alberto Tagliacollo, Victor Rennan Santos Ferreira, Wallace Beiroz, Yuri Kuikuro, Filipe Machado França

**Affiliations:** ^1^ Universidade Federal do Pará, Belém, Pará, Brazil; ^2^ School of Biological Sciences, University of Bristol, Bristol, UK; ^3^ Instituto Nacional de Pesquisas da Amazônia, Manaus, Amazonas, Brazil; ^4^ Universidade Federal do Amazonas, Manaus, Amazonas, Brazil; ^5^ Fundação Oswaldo Cruz, Rio de Janeiro, RJ, Brazil; ^6^ QuipoTech: Soluções Blockchain, Petrópolis, RJ, Brazil; ^7^ Universidade da Integração Internacional da Lusofonia Afro-Brasileira, Redenção, Ceará, Brazil; ^8^ Universidade de São Paulo, SP, Brazil; ^9^ University of Leeds, Leeds, UK; ^10^ Universidade Federal do Acre, Rio Branco, AC, Brazil; ^11^ Universidade Federal de São Carlos, São Carlos, SP, Brazil; ^12^ Instituto Militar de Engenharia, Rio de Janeiro, RJ, Brazil; ^13^ Universidade Federal do Amapá, Macapá, AP, Brazil; ^14^ Universidade Federal do Rio Grande do Norte, Natal, RN, Brazil; ^15^ Instituto Chico Mendes de Conservação da Biodiversidade, Brasília, DF, Brazil; ^16^ School of Geographical Sciences, University of Bristol, Bristol, UK; ^17^ University of Birmingham, Birmingham, UK; ^18^ Universidade Estadual de Feira de Santana, Feira de Santana, Bahia, Brazil; ^19^ Núcleo Regional de Roraima, Instituto Nacional de Pesquisas da Amazônia, Boa Vista, Roraima, Brazil; ^20^ Universidade Federal de Lavras, Lavras, MG, Brazil; ^21^ Lancaster University, Lancaster, UK; ^22^ Serviço Florestal Brasileiro, Ministério do Meio Ambiente e Mudança do Clima, Brasília, DF, Brasil, Brasília, Brazil; ^23^ Universidade Federal Rural da Amazônia, Belém, Pará, Brazil; ^24^ Instituto Tecnológico Vale, Belém, Pará, Brazil; ^25^ Centro de Inverstigação em Biodiversidade e Recursos Genéticos (CIBIO-InBIO), Portugal; ^26^ Universidade Federal de Mato Grosso, Cuiabá, MT, Brazil; ^27^ Naturalis Biodiversity Center, Leiden, Zuid-Holland, Netherlands; ^28^ Quantitative Biodiversity Dynamics, Biology, Utrecht University, Utrecht, Netherlands; ^29^ University of Louisiana at Lafayette, Lafayette, LA, USA; ^30^ Universidade Federal do Oeste do Pará, Santarém, Pará, Brazil; ^31^ Science Panel for the Amazon (SPA), Brazil; ^32^ Instituto Nacional de Áreas Úmidas, Brazil; ^33^ Biogeography Department, Universität Trier, Trier, Germany; ^34^ Universidade Federal do Pará, Altamira, Pará, Brazil; ^35^ Virginia Tech University, VA, USA; ^36^ Universidade Federal da Paraíba, João Pessoa, PB, Brazil; ^37^ Universidade Federal de Mato Grosso, Araguaia, Brazil; ^38^ Escola Superior de Agricultura Luiz de Queiroz, Universidade de São Paulo, Piracicaba, SP, Brazil; ^39^ Secretaria de Estado de Meio Ambiente e Sustentabilidade do Pará, Belém, Pará, Brazil; ^40^ Universidade Federal de Goiânia, Goiânia, GO, Brazil; ^41^ Universidade de Brasília, Brasília, DF, Brazil; ^42^ Museu Paraense Emílio Goeldi, Coordenação de Ciências da Terra e Ecologia, Belém, PA, Brazil; ^43^ Universidade Federal de Uberlândia, Uberlândia, Brazil; ^44^ Universidade do Estado de Mato Grosso, Nova Xavantina, MT, Brazil

**Keywords:** Brazilian National Institute of Science and Technology, research investments, tropical biodiversity, gender equality, collaborative research networks

## Abstract

The Amazon region is critical for maintaining global biodiversity and mitigating climate change; however, it faces escalating threats from deforestation and habitat degradation. Addressing these threats requires evidence-based strategies grounded in investments in science, technology, innovation and collaborative research. The Brazilian National Institute of Science and Technology (INCT) programme plays a central role in advancing scientific and technological progress by establishing collaborative research networks across diverse fields and regions. In this context, we present the INCT in Synthesis of Amazonian Biodiversity (INCT-SynBiAm) as a case study, illustrating how research networks can promote diversity in academia and enhance our understanding of biodiversity in hyperdiverse tropical regions. The SynBiAm network integrates 47 academic and non-academic institutions from Brazil and abroad. Its key objectives are to establish and expand a collaborative initiative for research synthesis in Amazonia, deepen our understanding of biodiversity patterns, threats and drivers in forest and freshwater ecosystems, inform environmental and educational practices and policies, and train future educators, decision-makers and scientists committed to the Amazon’s conservation and sustainability. We outline the INCT programme and demonstrate how the INCT-SynBiAm network can achieve these goals, providing a model for future collaborative initiatives aimed at addressing socio-ecological challenges in tropical regions.

## 1. Introduction

The loss of biodiversity and the climate crisis represent two of society’s most pressing global challenges [1,2]. In response, governments worldwide are increasingly investing in technologies and strategies aimed at protecting biodiversity [3], restoring ecosystems [4] and promoting climate adaptation and mitigation [5]. Addressing these challenges requires the rapid development and implementation of technologies, environmental policies and practices informed by scientific evidence [6,7]. Understanding the interconnections between biodiversity loss and climate change is crucial for assessing the risk of large-scale tipping points and enhancing ecosystem resilience [8,9]. This is particularly important for ecoregions exhibiting high environmental heterogeneity and socio-ecological diversity, such as the Amazon. However, limited information on Amazonian biodiversity and ecosystem dynamics—compounded by under-sampled areas and research biases [10]—creates significant knowledge gaps that hinder our ability to develop evidence-based conservation strategies. In this context, collaborative research networks can help bridge these gaps by strengthening research infrastructure, improving data accessibility and quality and enhancing biodiversity monitoring [11–13].

Brazil hosts more than 60% of the Amazon River Basin and the largest remaining tropical forest on the planet. At the same time, it is home to over 212 million people [14] and one of the world’s largest producers and/or exporters of many agricultural commodities [15], hydropower electricity [16] and minerals [17]. This represents a significant challenge for balancing development and environmental conservation in the country. In the Brazilian Amazon, increased deforestation rates, forest fires, poaching, illegal exploitation of natural resources, extreme climate events and declining precipitation have led to uncertainties for the future of ecosystems and the people who depend on them [18–21]. Recent socio-political and environmental turmoil has raised concerns about the future of Brazilian science (e.g. [22,23]) and its path towards sustainability [24]. It is therefore crucial to ensure that decision-making and policy-making in Amazonia are grounded in scientific evidence while accounting for the region’s diverse socio-environmental context. Collaborative research networks, which involve not only scientists but also practitioners, decision-makers, Indigenous peoples and local communities—who possess a practical and diverse body of knowledge—are essential in effectively addressing biodiversity and ecosystem challenges [25,26]. By engaging these communities, such networks can enhance our understanding of the solutions that need to be prioritized and strengthen local and regional capacities for protecting biodiversity and restoring ecosystems [13,27–29].

Although numerous initiatives (e.g. Amazon Tree Diversity Network [30] and PREDICTS [31]) focus on local and global biodiversity, many face significant methodological, geographic and taxonomic challenges. This is because quantifying biodiversity with standardized methods is costly and labour-intensive [32,33], making accessibility, research infrastructure and data availability key limitations for assessing tropical biodiversity in remote regions and at macroecological scales [10,34]. Collaborative research networks and biodiversity studies are also constrained by ecosystem and geographic limitations. For instance, the tropics have been neglected in ecology and evolution research [35–37], while the coordination of collaborative research networks and databases focused on tropical biodiversity is historically located in Global North countries—where research funding and infrastructure are more abundant—and has poor gender representation as well as of scientists from Latin America and other Global South regions [38–42]. Conservation efforts and global biodiversity databases are often taxonomically biased towards plants and vertebrates, providing much less attention to invertebrates—which represent most of the biodiversity on Earth [43]. This bias is evidenced by the fact that only around 1.2% (12 747 of >1 million) of the currently known insect species have had their conservation status assessed by the IUCN, compared witho 84% of vertebrates and 17% of plants [44]. This raises concerns given the expected extinction rates and biodiversity declines driven by climate change and other anthropogenic threats happening across the tropics, particularly in Amazonia [45–48]. Finally, although scientific research has expanded across the tropics, in-country funding distribution inequalities [49], lack of integration of local communities and knowledge systems into the scientific process [50], science-policy gaps and institutional constraints [51] can limit the development and implementation of evidence-based policy and decision-making in tropical countries.

In this context, we present the National Institute of Science and Technology (INCT) programme, with a particular focus on the ‘Synthesis of Amazonian Biodiversity’ (INCT-SynBiAm or INCT-SinBiAm (in Portuguese)) Network—a recently established collaborative and multidisciplinary network. Our overall aim is to argue for the importance of investments in collaborative research and networks to enhance our knowledge of tropical biodiversity and promote inclusive research environments. Specifically: (i) we assess inequalities in research investments on biodiversity and gender representation within the INCT programme to contextualize opportunities and challenges for Amazonian research; (ii) we use the INCT-SynBiAm conceptual and methodological framework as a case study to inform and foster other similar initiatives across the tropics; and (iii) we present proposed tools, actions and partnerships developed by SynBiAm. Furthermore, we share lessons learnt from the development of our collaborative research network and propose potential future directions through which the Brazilian INCT programme and affiliated research networks could achieve a long-lasting impact on shaping future biodiversity research, conservation practices, and environmental policies.

## 2. The Brazilian National Institute of Science and Technology programme

### (a) Overview

The INCT programme was established in 2008 as a national initiative supporting strategic research areas critical to Brazil’s development. Each INCT network represents an individual research institute funded through this programme. Throughout this paper, we refer to the whole national programme as ‘INCT programme’; to the individual institutes as ‘INCT(s)’ and/or ‘INCT network(s)’; and our network focused on Amazonian biodiversity synthesis as ‘INCT-SynBiAm’. The INCT programme is coordinated by the Brazilian Ministry of Science, Technology and Innovation, in partnership with other national agencies responsible for research, higher education and health policy—including the National Council for Scientific and Technological Development (CNPq). The programme supports a network of INCTs, representing a cornerstone initiative to foster scientific and technological excellence across a broad spectrum of epistemic fields and strategic areas. The programme’s primary goals include developing and training human resources, exchanging knowledge and technologies, supporting research and development, and integrating Brazilian research into the global scientific context. The INCT programme also plays a pivotal role in scientific education and outreach by establishing environments that attract, retain and train students and early-career researchers. Through funded networks, the INCT programme advances basic and applied research while strengthening Brazil’s scientific infrastructure. This includes establishing laboratories and providing support to institutions nationwide, thereby laying a robust foundation for scientific progress.

### (b) Research investments on biodiversity and gender representation within the National Institute of Science and Technology programme

Since 2008, the INCT programme has invested over R$2.5 billion Brazilian reais (approx. 500 million USD) through five calls for applications, supporting 324 INCT networks (see details in electronic supplementary material, table S1). These networks cover a wide range of disciplines, from botany, ecology and zoology (hereafter biodiversity-related fields) to nanotechnology, public health and renewable energy. The programme has made significant investments in biodiversity-related research. Using publicly available data from the CNPq Data Panel [52], we found a total of 228 scholarships funded through the INCT programme from 2016 to 2023 for biodiversity-related fields. These scholarships include technical research support (*n* = 79), training of undergraduate students (*n* = 71), industrial and technological development (*n* = 56), postdoctoral positions (*n* = 16) and other categories (*n* = 6), which underscores the programme’s emphasis on capacity building (see electronic supplementary material, table S2). While this investment might seem modest given the magnitude of the country’s biodiversity and environmental challenges, it remains one of the few national mechanisms designed to promote long-term scientific collaboration and coordinated capacity building in Brazil, especially in underrepresented regions such as the Amazon.

**Figure 1.**
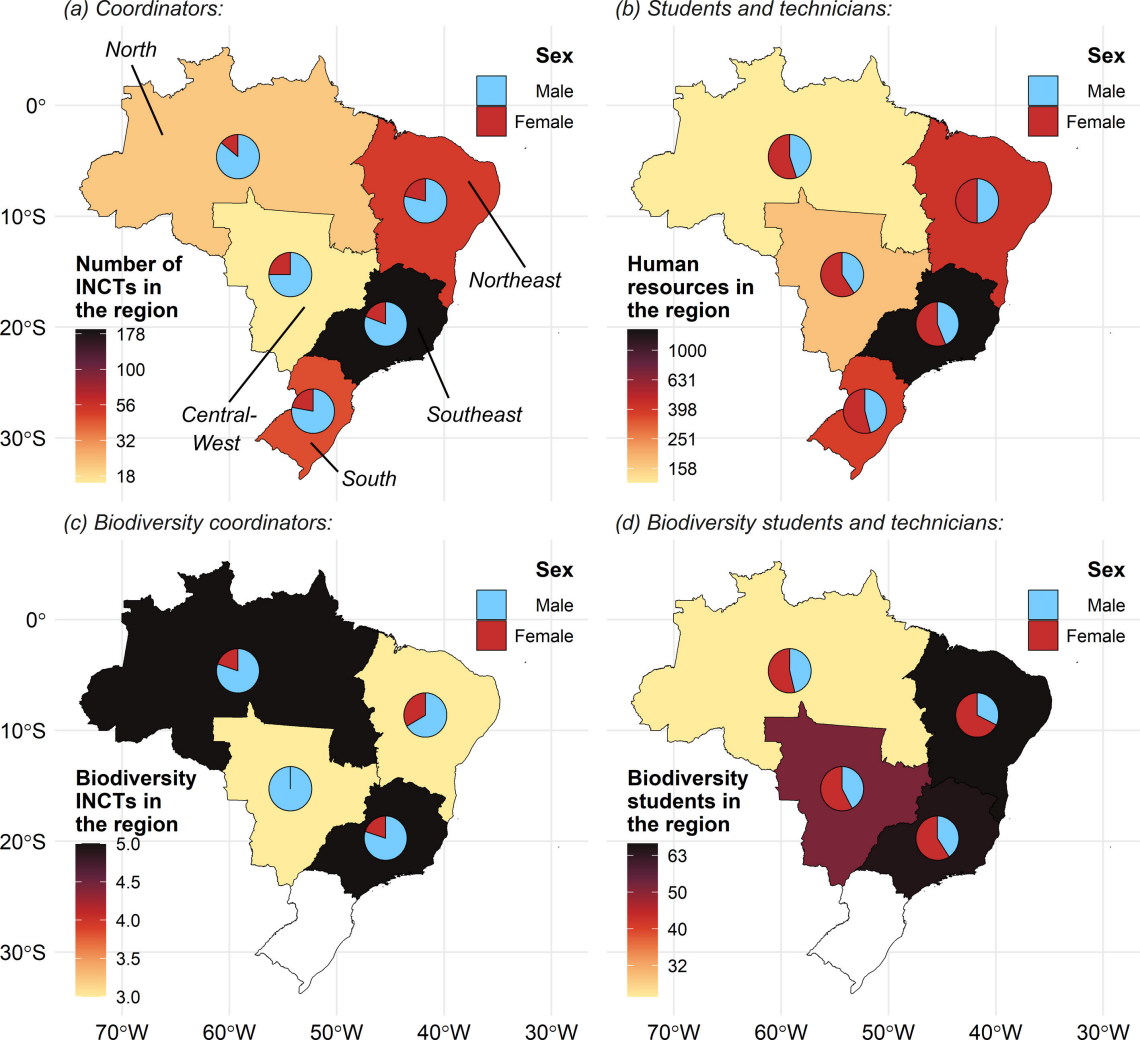
Regional and gender distribution of funding across the Brazilian INCT programme (*a*,*b*) and for biodiversity-related projects funded by the INCT programme (*c*,*d*). The number of coordinators, students and technicians for biodiversity-related INCTs was not available for the South region. Human resources (*b*) and biodiversity students (*d*) are based on scholarship numbers provided by the INCT programme at distinct levels between 2016 and 2023 (see electronic supplementary material, tables S2 and S3). Biodiversity-related INCTs account for investments and scholarships within the botany, ecology and zoology fields. All data are publicly available and were extracted from the CNPq Data Panel [52].

Apart from the differences between the individual calls, INCT programme research investments also vary across Brazilian regions (figure 1). Since 2008, the Southeast region has received the highest number of funded networks (*n* = 191) and scholarships (*n* = 71), followed by the Northeast (*n* = 51 networks and 71 scholarships), South (*n* = 45 networks, number of scholarships not available), North (*n* = 21 networks and 31 scholarships) and Central-West regions (*n* = 16 networks and 55 scholarships; figure 1; electronic supplementary material, table S3). Although all the 13 INCTs currently active in the North region are concentrated in just two municipalities (Manaus and Belém), most of these networks are specifically focused on Amazonian biodiversity (*n* = 7; see electronic supplementary material, table S4). This distribution of INCTs and scholarships underscores historical asymmetries in infrastructure, institutional capacity and access to national funding mechanisms—both within and between the Brazilian regions (e.g. [49]).

Inequalities extend beyond the distribution of resources, as evidenced by the under-representation of women in the coordination of INCT networks (figure 1). Female representation reached up to 25% among regional coordinators of INCT networks (figure 1*a*)—with only 20% of biodiversity-related INCTs in the North region being led by women (figure 1*c*). Gender representation was more evenly distributed among students and technical staff, both at the national programme and for biodiversity-focused INCTs (figure 1*b*,*d*). While reflecting the patterns of inequalities in academia across various countries and disciplines [42,53], it highlights the critical need for targeted actions and policies to ensure diverse representation in science and to address the intersections of regional and gender disparities in research funding [42,54].

## 3. The National Institute of Science and Technology in Synthesis of Amazonian Biodiversity network: an Amazonian case study on ecological synthesis research

Synthesis research plays a crucial role in consolidating knowledge in various fields and provides valuable insights for decision-making at multiple levels [28]. Globally, synthesis research has become increasingly important for understanding large-scale biodiversity patterns, identifying drivers of change and addressing complex socio-ecological challenges [48,55,56]. In Brazil, synthesis research has been relatively underdeveloped compared to Global North countries, yet it is essential for guiding long-term conservation efforts, sustainable management strategies and socio-ecological policy decisions (e.g. [28,57–60]). Within Brazil, even the relatively few initiatives that do exist are mostly based outside Amazonia and focused on synthesizing the knowledge of non-Amazonian biodiversity (e.g. Biota Synthesis, https://biotasintese.iea.usp.br [57]).

The INCT-SynBiAm network was officially launched in December 2023 during an in-person meeting held in Belém, Pará, with the participation of approximately 70 members from various institutions and regions. Funded for an initial 5-year period, the network builds on previous collaborations and aims to consolidate synthesis research on Amazonia biodiversity. Its governance structure includes a steering committee with four coordinators and seven thematic working groups, each coordinated by two researchers. Members were selected based on scientific expertise, regional and gender representation, prior collaborations and active engagement in biodiversity science and policy. Additionally, during the proposal submission, we mapped and identified potential new members (both academics and non-academics) with expertise in key topics for the network (e.g. policy, decision-making, science communication, and engagement with traditional communities).

**Figure 2.**
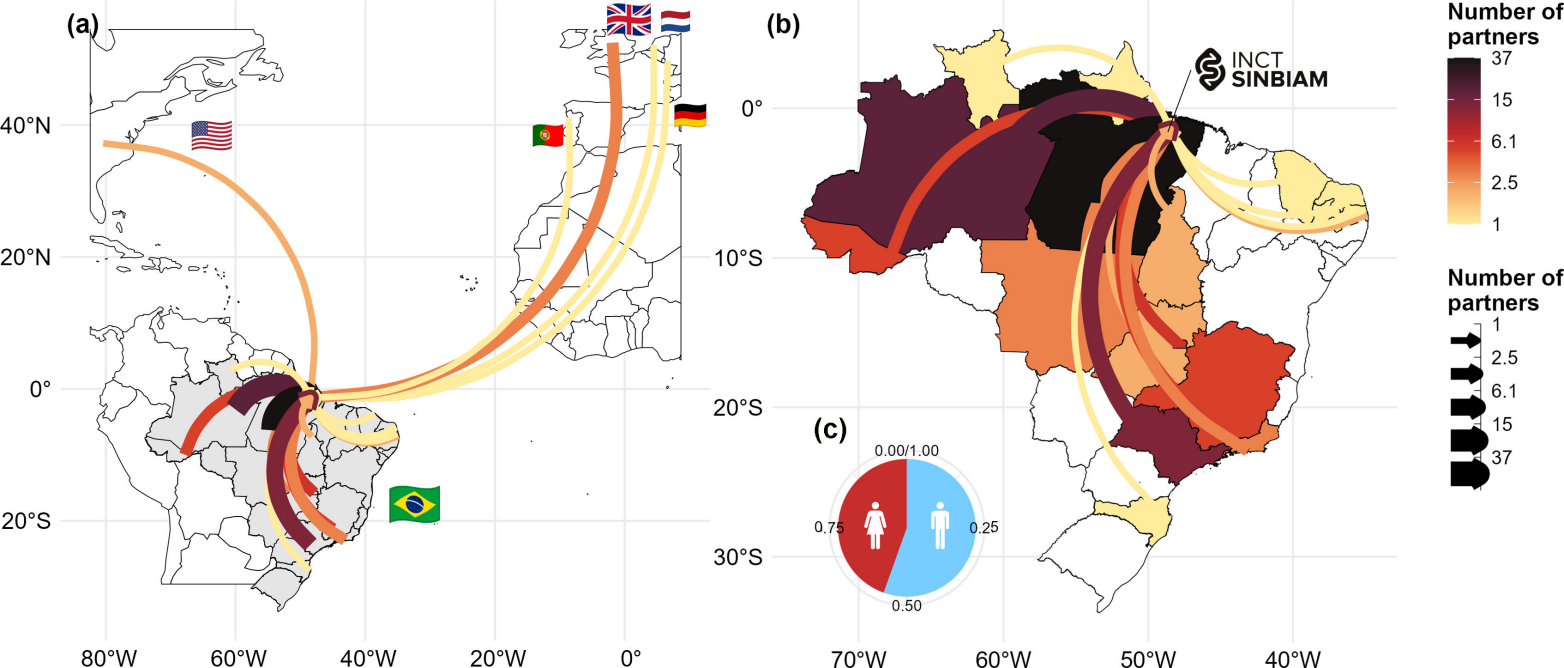
Spatial distribution of scientists affiliated with INCT-SynBiAm (a) across the globe and (b) within Brazil. In panels (a) and (b), the colour and thickness of the connecting lines indicate the number of collaborators in each country or state, all converging toward the INCT-SynBiAm headquarters in the eastern Amazon. In panel (b), Brazilian states without fill colour have no affiliated members, while coloured states reflect varying numbers of partners. Panel (c) shows the gender distribution of all affiliated members, with red indicating women and blue indicating men.

Currently, the INCT-SynBiAm has 110 members from 47 institutions, 39 in Brazil and 8 abroad. Our members and institutions are distributed across the Brazilian regions, including 67 members and 17 institutions in the North, 22 members and 12 institutions in the Southeast, 4 members and 3 institutions in the Northeast, 9 members and 6 institutions in the Central West, and 1 member and 1 institution in the South (figure 2a). SynBiAm’s membership also varies across sectors and genders (figure 2b). Female scientists represent 44.5% (*n* = 49) of the network members and 50% (*n* = 8) of the leadership positions (figure 2c). Regarding the career-level distribution, INCT-SinBiAm has 22 postdoctoral researchers, 8 graduate students, 53 university faculty members, 3 undergraduate students, 7 academic researchers and 17 non-academic members. In Brazil, members are geographically distributed across 28 cities in 17 states. Outside Brazil, members are present across five countries (England, Portugal, the Netherlands, the United States and Germany).

Our network exemplifies the power of collaborative science to tackle complex biodiversity challenges and provides a model for future initiatives across the tropics. The INCT-SynBiAm network was established to synthesize existing knowledge on Amazonian forest and freshwater biodiversity and generate relevant scientific evidence to tackle local and regional environmental challenges through three overarching goals: (i) strengthening and expanding a collaborative research network focused on synthesizing data on Amazonian terrestrial and freshwater biodiversity data, (ii) informing practices and public policies related to education, conservation and sustainable management, and (iii) promoting the training of new generations of decision-makers, educators and scientists working in the Amazon. To achieve this, we expanded a previous network built through investments from the Brazilian Synthesis Centre on Biodiversity and Ecosystem Services (SinBiose/CNPq)—one of the first Global South-based synthesis research centres focused on ecological studies [57]—through the project ‘SYNthesising Ecological Responses to deGradation In amaZonian Environments’ (see [10]). In doing so, INCT-SynBiAm brought together scientists, practitioners, policy-makers, decision-makers and community leaders to build a multidisciplinary network and participatory approaches to fill gaps in ecological synthesis research.

Aiming to overcome structural barriers, INCT-SynBiAm has implemented deliberate actions to foster greater equity in both regional and gender representation. For example, 45% of its members are women, while 67 of its members and 17 of its institutions are based in the Amazonian region, which has been historically underrepresented in research funding for biodiversity in Brazil (figure 2) [49]. The choice of the leading and member institutions was driven by recognizing the role of Amazonian institutions to lead capacity-building efforts aligned with local realities while promoting the within-country and international collaborations with partners from other Brazilian regions and countries. For instance, INCT-SynBiAm collaborates with several other INCTs and research network initiatives. Beyond collaboration, SynBiAm plays a central role in synthesizing biodiversity knowledge, acting as a hub for integrating and organizing field data generated by researchers from and beyond our network. In this way, the institute currently acts as a ‘network of networks’ (or ‘meta-network’) aiming to enhance the collective impact of biodiversity research in the region. As a result of these collaborations, the network has already led to 12 peer-reviewed scientific publications (electronic supplementary material, table S5) and reached out to over 1000 people through the (i) organization of symposiums and meetings, (ii) design of educational materials, and (iii) implementation of participatory workshops involving local communities, educators and environmental managers. These efforts reflect the network’s aim to bridge scientific knowledge with conservation and education practices across different regions of the Amazon.

## 4. Developed approaches and learnt lessons to enhance tropical biodiversity knowledge

### (a) TAOCA: a technological tool to store and validate Amazonian biodiversity data

Biodiversity databases play a pivotal role in biodiversity conservation and monitoring efforts. These initiatives enable efficient and long-term storage, sharing and analysis of large amounts of data while promoting international collaborations and providing a solid foundation for developing evidence-based public policies [61–64]. Biodiversity databases also enhance data transparency and standardization, support environmental education and foster public access and participation in data collection and conservation efforts [65,66]. Among global initiatives, the Global Biodiversity Information Facility (GBIF) is the world’s leading biodiversity database, currently storing over 3 billion occurrence records (https://www.gbif.org, accessed January 2025). Despite all the currently available information, ongoing shortfalls in species taxonomy, abundance and spatial distribution still limit our understanding of biodiversity patterns, particularly in the tropics [67–71]. Moreover, there is a critical gap in standardized sampling effort data, which hinders not only the analysis of species occurrence, but also robust estimates of abundance and population trends over time.

**Figure 3.**
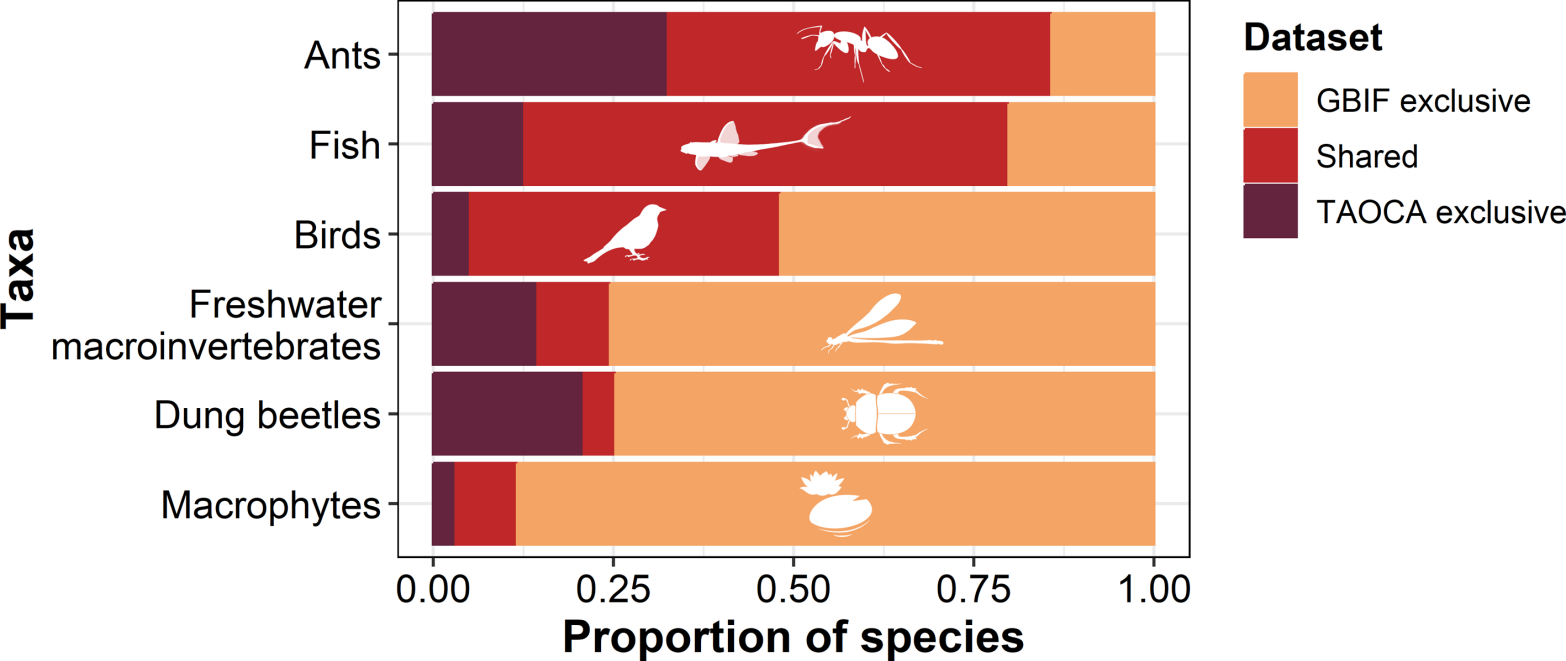
Exclusive and shared species records for ants, fishes, birds, freshwater macroinvertebrates, dung beetles and macrophytes between the GBIF and TAOCA databases. Assessed in January 2025.

To fill these gaps and achieve our mission of understanding the patterns of Amazonian biodiversity, members of the INCT-SynBiAm Network co-founded the TAOCA (acronym for ‘Traits, Abundances, Occurrences, and Communities from Amazonia’) database (for details, see https://www.taoca.net), which integrates, curates and stores raw data on Amazonian forest and freshwater biodiversity. TAOCA employs machine learning models to validate and curate taxonomy and geographic information, with the goal of ensuring that the biodiversity data is taxonomically updated and available for the scientific and practitioner communities in the longer term. Currently, it stores 922 234 records from 6834 (morpho)species (accessed January 2025), including ants (*n* = 1148), birds (*n* = 855), dung beetles (*n* = 444), freshwater macroinvertebrates (e.g. Ephemeroptera, Plecoptera, Trichoptera, Odonata and Hemiptera; *n* = 881), macrophytes (*n* = 724) and fishes (*n* = 2782) sampled from forest and freshwater environments within Brazil’s Legal Amazon states. These records derive from raw datasets collected and provided by over 500 data contributors. When comparing Amazonian species records from TAOCA and GBIF (figure 3), we found that the overlap between databases varied across taxa: 67% for fishes, 54% for ants, 43% for birds, 10% for freshwater insects, 9% for macrophytes and 5% for dung beetles. Our assessment also highlights the number of exclusive species records for both databases (figure 3). TAOCA hosts exclusive species records ranging from 3% (for macrophytes) to 32% (for ants), while GBIF includes exclusive records ranging from 14% (for ants) to 88% (for macrophytes) of species occurring in the Amazonian region.

TAOCA is the first and currently one of the most comprehensive online databases that systematically compiles standardized ecological data on the terrestrial fauna of the Brazilian Amazon. Similar to other large-scale biodiversity databases (e.g. ForestPlots [72] and TreeCo [73] for trees; the Global Ants Database [74] for ants; and PREDICTS [31] for multiple taxa), TAOCA enables large-scale, high-quality data analysis for multiple species. The main advances of TAOCA are as follows: (i) the integration of raw species abundance data collected using standardized and documented methods (e.g. GBIF focuses primarily on species abundance data obtained from various sampling methods); (ii) the integration of data from forest and freshwater ecosystems as well as from multiple biological groups (e.g. many previous initiatives have focused on a particular ecosystem and/or taxa when integrating data from different species); and (iii) the leading role of local institutions in decision-making and management of the datasets aggregated in the database (e.g. most initiatives storing global and tropical biodiversity data are based in the Global North and not in the tropics). In addition, TAOCA is currently being structured to include datasets from other Amazonian countries, further increasing its global relevance and importance for tropical biodiversity research. Ensuring TAOCA’s long-term sustainability and funding is a key priority for our network. Our current strategies include: (i) integration with public platforms such as SiBBr (Brazilian Biodiversity Information System, https://www.sibbr.gov.br), which are highly relevant for environmental policy and decision-making in Brazil; (ii) collaborative governance with scientific networks, institutional partners and data providers; (iii) ongoing support from computer scientists and programmers for technical development and data security; and (iv) securing additional funding via national and international grants. These measures aim to preserve and expand TAOCA as a reference platform for Amazonian biodiversity.

### (b) Intersectoral and international collaborations

Intersectoral and international partnerships play a crucial role in advancing science, particularly within research networks, as they address the following two key aspects. (i) *Promoting a transdisciplinary approach and knowledge exchange* by involving stakeholders from governmental, business, academic, NGO and civil society sectors can facilitate the integration of diverse perspectives into the scientific process [75]. This integration fosters innovative and effective solutions through the sharing of knowledge and best practices, enriching research by adapting and advancing scientific methods within different cultural and sectoral perspectives [76]. (ii) *Enhancing accessibility to resources and impact*: partnerships with the public sector and non-governmental organizations can increase research uptake by decision-makers and policy-makers. Collaborating with the private sector provides funding opportunities and technology exchanges. International partnerships promote knowledge exchanges and access to state-of-the-art infrastructure and specialized equipment, often not available in some regions or countries. Intersectoral partnerships can also enhance the legitimacy of scientific research by demonstrating a collaborative and inclusive approach, which is essential to ensure that research outcomes are translated into evidence-based decision-making and public policies [77,78].

As an interdisciplinary and inter-institutional network that includes both public and private initiatives, the INCT-SynBiAm aligns with these points because the network is composed of members from different sectors, including park managers, environmental decision-makers, community leaders and researchers from Brazilian and international institutions, and a technology-based company focused on developing environmental solutions (QuipoTech, https://www.quipotech.com)—which has been directly involved in the TAOCA creation, maintenance and expansion. The broad representation of members across various sectors enhances the network’s credibility and legitimacy when proposing information for use in public policies.

### (c) Knowledge co-production

Knowledge co-production represents a transformative approach in research [79]. It can foster intersectoral and multidisciplinary collaborations, as well as the integration of scientific, Indigenous and local knowledge systems. In doing so, knowledge co-production promotes inclusivity and innovative problem-solving, including holistic and contextually relevant interventions in biodiversity and ecosystem conservation [25,80,81]. A key strength of knowledge co-production lies in bridging the research-practice and research-policy gaps [82,83]. Through stakeholder engagement and participatory approaches, it can generate and enhance the uptake of scientific evidence, often leading to solutions of complex challenges while aligning research with societal needs [28,84–86]. Knowledge co-production has also been highlighted as a pathway to effective environmental management and governance [85], while the adoption of participatory approaches can lead to conservation strategies that are ethically grounded and aligned with the local socio-ecological context and needs [87,88]. By transcending disciplinary niches, knowledge co-production can lay the foundation for sustainable and inclusive development and proactive biodiversity research and conservation [81,88,89].

The INCT-SynBiAm is promoting and encouraging co-production of knowledge and scientific evidence with traditional people, policy-makers, decision-makers and practitioners. We engage with these diverse sectors of society through participatory approaches and initiatives that also promote capacity building and science communication. Co-production approaches are also adopted across different levels of the educational system in Brazil. For example, bringing together secondary school teachers, Indigenous and non-Indigenous researchers, and leaders from Indigenous and traditional communities to co-design biodiversity-focused educational materials and digital tools, as well as training university students through pedagogical/teaching residencies at rural and Indigenous schools, which is aligned with Brazil’s Pedagogical Residency Programme (for details, see [90]).

Since its launch in late 2023, SynBiAm has supported engagement and capacity-building activities across five states in the Brazilian Amazon. These initiatives have included citizen science workshops, participatory biodiversity monitoring, and capacity building in data management and field protocols. In total, over 500 individuals have benefited from these efforts, including students, local leaders, traditional and Indigenous community members, and early-career researchers. A notable example of SynBiAm’s impact is the participatory biodiversity monitoring programme conducted with the Panará Indigenous people in Mato Grosso, which began in early 2024. This initiative trained 26 Panará youth leaders in data collection using simplified protocols to survey aquatic insects and water indicators. The activity was co-designed with community representatives and conducted in their territory, promoting autonomy in environmental monitoring. Such efforts were previously rare due to the lack of targeted funding and logistical support. SynBiAm provided the financial and human resources, technical coordination and institutional articulation required to overcome previously existing barriers. Another important activity of the network was supporting the 7th Symposium on Neotropical Aquatic Insects. This event gathered approximately 400 participants and resulted in a collaborative article with the researchers involved, presenting a synthesis of the key findings and discussions from the symposium. Thanks to the support provided by INCT-SynBiAm, registration fees were reduced, which facilitated hosting the event in the Amazon region and ensured strong participant representation. For further details on INCT-SynBiAm activities and impact, see electronic supplementary material, table S6.

### (d) Retention and training of early-career researchers

The retention of a critical mass of high-skilled investigators is a fundamental aspect of scientific advancement and effective research [91]. Investing in the education of qualified scientists and creating opportunities to keep these professionals is essential for strengthening research and innovation infrastructure, particularly in the Global South where brain drain is a common phenomenon [92,93]. Research networks can promote the retention of early-career researchers by enhancing their scientific impact [94], performance [95,96] and citability of their work [97,98]. By having embedded experiences and local knowledge, these scientists can have a greater policy relevance [99] and an impact on governance performance [100]. When scientists remain in their local institutions, they also play a key role in training the future generations at various levels—undergraduate, graduate or specialization, which is essential to foster innovation and technological development in the long term. In INCT-SynBiAm, non-academics (*n* = 17) and early-career researchers (e.g. postgraduate and postdocs; *n* = 30) account for approximately 42% of our members. In terms of human resource development, our network currently supports 21 scholarship holders, including eight undergraduate researchers, six postdoctoral fellows and seven scholars focused on Technological and Industrial Development. These scholarships aim to strengthen local capacity and foster the next generation of scientists and practitioners engaged in Amazonian biodiversity research and policy.

Collaborative research networks can also promote science communication and training beyond the academic world. For example, our network has created opportunities for early-career researchers to engage with local schools and develop educational materials (e.g. e-books, digital tools and online scientific content) targeting different audiences and educational groups and levels. Our goal is to both train early-career scientists in science communication and produce valuable tools to empower educators and inspire students to engage more with conservation, science, decision-making and policy in Amazonia. Despite its early achievements, SynBiAm faces structural and operational challenges common to research networks in tropical regions. First, funding distribution inequalities affect scientific infrastructure across institutions and regions within the INCT programme (figure 1), with many leading institutions and resources located outside the Amazon region. This poses logistical and equity-related barriers to capacity-building and community engagement. The network also operates under time-limited funding, which constrains long-term planning and hinders the development of long-lasting partnerships with local governments and civil society. Furthermore, the articulation between academic, governmental and non-governmental stakeholders remains uneven across regions, with most non-academic members being based in Manaus and Belém—the two largest cities in Amazonia. Recognizing these challenges is essential to guide future improvements and ensure the network’s sustainability.

## 5. The National Institute of Science and Technology in Synthesis of Amazonian Biodiversity—conclusion and future perspectives

Our experiences emphasize the critical importance of fostering a collaborative network of researchers, policy-makers, traditional people and local communities within and about Amazonia. Such interconnected, multidisciplinary and intersectoral efforts are vital not only for protecting the unique biodiversity of tropical regions but also for supporting practices and policies that balance environmental conservation with the well-being and quality of life of local communities. By integrating diverse knowledge systems, collaborative research networks have a great potential to co-develop pathways and solutions to tackle the socio-ecological challenges faced by tropical regions [26].

Looking ahead, the INCT-SynBiAm envisions expanding its contributions by generating and making available more ecological data and scientific knowledge to inform environmental practices and public policies. Our future initiatives will prioritize amplifying the voices of traditional peoples, ensuring their knowledge is respected and integrated into the scientific process and outputs. Furthermore, we aim to train a new generation of students and researchers to continue advancing science in the Amazon, fostering long-term regional development and innovation. To achieve these goals, increased and sustained investments in research on Amazonian biodiversity will be essential [49]. The region still houses multiple ecological knowledge gaps to be investigated [10], and its sustainable development and biodiversity conservation, as well as those in other tropical regions, are crucial for mitigating the current biodiversity and climate crises.

## Data Availability

All datasets and scripts used in this study are available on Zenodo [101]. Further results can be found in the electronic supplementary material. Supplementary material is available online [102].

## References

[1] Pecl GT *et al*. 2017 Biodiversity redistribution under climate change: impacts on ecosystems and human well-being. Science **355**, eaai9214. (10.1126/science.aai9214) 28360268

[2] Rinawati F, Stein K, Lindner A. 2013 Climate change impacts on biodiversity: the setting of a lingering global crisis. Diversity **5**, 114–123. (10.3390/d5010114)

[3] Firn J *et al*. 2015 Priority threat management of invasive animals to protect biodiversity under climate change. Glob. Chang. Biol. **21**, 3917–3930. (10.1111/gcb.13034) 26179346

[4] Bustamante MMC *et al*. 2019 Ecological restoration as a strategy for mitigating and adapting to climate change: lessons and challenges from Brazil. Mitig. Adapt. Strateg. Glob. Change **24**, 1249–1270. (10.1007/s11027-018-9837-5)

[5] Stern PC, Dietz T, Nielsen KS, Peng W, Vandenbergh MP. 2023 Feasible climate mitigation. Nat. Clim. Chang. **13**, 6–8. (10.1038/s41558-022-01563-7)

[6] Fawzy S, Osman AI, Doran J, Rooney DW. 2020 Strategies for mitigation of climate change: a review. Environ. Chem. Lett. **18**, 2069–2094. (10.1007/s10311-020-01059-w)

[7] Alestra C, Cette G, Chouard V, Lecat R. 2024 How can technology significantly contribute to climate change mitigation? Appl. Econ. **56**, 4925–4937. (10.1080/00036846.2023.2227416)

[8] Flores BM *et al*. 2024 Critical transitions in the Amazon forest system. Nature **626**, 555–564. (10.1038/s41586-023-06970-0) 38356065 PMC10866695

[9] França FM *et al*. 2020 Climatic and local stressor interactions threaten tropical forests and coral reefs. Phil. Trans. R. Soc. B **375**, 20190116. (10.1098/rstb.2019.0116) 31983328 PMC7017775

[10] Carvalho RL *et al*. 2023 Pervasive gaps in Amazonian ecological research. Curr. Biol. **33**, 3495–3504. (10.1016/j.cub.2023.06.077) 37473761

[11] Pereira HM, David Cooper H. 2006 Towards the global monitoring of biodiversity change. Trends Ecol. Evol. **21**, 123–129. (10.1016/j.tree.2005.10.015) 16701487

[12] Caudron A, Vigier L, Champigneulle A. 2012 Developing collaborative research to improve effectiveness in biodiversity conservation practice. Journal of Applied Ecology **49**, 753–757. (10.1111/j.1365-2664.2012.02115.x)

[13] Adams J. 2012 The rise of research networks. Nature **490**, 335–336. (10.1038/490335a) 23075965

[14] IBGE. 2024 Brazil’s population reaches 212.6 million. See https://www.gov.br/secom/en/latest-news/2024/08/ibge-brazils-population-reaches-212-6-million.

[15] Valdes C. 2022 Brazil’s momentum as a global agricultural supplier faces headwinds. See https://www.ers.usda.gov/amber-waves/2022/september/brazil-s-momentum-as-a-global-agricultural-supplier-faces-headwinds.

[16] Zarfl C, Lumsdon AE, Berlekamp J, Tydecks L, Tockner K. 2015 A global boom in hydropower dam construction. Aquat. Sci. **77**, 161–170. (10.1007/s00027-014-0377-0)

[17] Global Business Reports. 2023 Brazil mining report. See https://www.gbreports.com/files/pdf/_2023/Brazil-Mining-2023-web.pdf (accessed 4 November 2025).

[18] Fonseca MG, Alves LM, Aguiar APD, Arai E, Anderson LO, Rosan TM, Shimabukuro YE, Cruz de Aragão LEO. 2019 Effects of climate and land‐use change scenarios on fire probability during the 21st century in the Brazilian Amazon. Glob. Chang. Biol. **25**, 2931–2946. (10.1111/gcb.14709) 31304669

[19] França F, Solar R, Lees AC, Martins LP, Berenguer E, Barlow J. 2021 Reassessing the role of cattle and pasture in Brazil’s deforestation: A response to ‘Fire, deforestation, and livestock: When the smoke clears’. Land Use Policy **108**, 105195. (10.1016/j.landusepol.2020.105195)

[20] Santos de Lima L, Oliveira e Silva FE, Dorio Anastácio PR, Kolanski MMP, Pires Pereira AC, Menezes MSR, Cunha E, Macedo MN. 2024 Severe droughts reduce river navigability and isolate communities in the Brazilian Amazon. Commun. Earth Environ **5**, 1–12. (10.1038/s43247-024-01530-4)

[21] Silva RM da, Lopes AG, Santos CAG. 2023 Deforestation and fires in the Brazilian Amazon from 2001 to 2020: Impacts on rainfall variability and land surface temperature. J. Environ. Manage. **326**, 116664. (10.1016/j.jenvman.2022.116664) 36370609

[22] Escobar H. 2021 Brazilian scientists lament ‘freeze’ on research budget. Science **364**. (10.1126/science.aax6227) 30975866

[23] de Oliveira Andrade R. 2024 Brazil’s plummeting graduate enrolments hint at declining interest in academic science careers. Nature **630**, 518–519. (10.1038/d41586-024-01504-8) 38773310

[24] Dobrovolski R, Loyola R, Rattis L, Gouveia SF, Cardoso D, Santos-Silva R, Gonçalves-Souza D, Bini LM, Diniz-Filho JAF. 2018 Science and democracy must orientate Brazil’s path to sustainability. Perspect Ecol Conserv **16**, 121–124. (10.1016/j.pecon.2018.06.005)

[25] da Silva EC, Guerrero-Moreno MA, Oliveira FA, Juen L, de Carvalho FG, Barbosa Oliveira-Junior JM. 2025 The importance of traditional communities in biodiversity conservation. Biodivers. Conserv. **34**, 685–714. (10.1007/s10531-024-02999-3)

[26] Nóbrega RLB *et al*. 2023 Co-developing pathways to protect nature, land, territory, and well-being in Amazonia. Commun. Earth Environ **4**, 1–5. (10.1038/s43247-023-01026-7)37325084

[27] Barlow J *et al*. 2011 Using learning networks to understand complex systems: a case study of biological, geophysical and social research in the Amazon. Biol. Rev. Camb. Phil. Soc. **86**, 457–474. (10.1111/j.1469-185X.2010.00155.x) 20849493

[28] Metzger JP *et al*. 2024 Guiding transdisciplinary synthesis processes for social-ecological policy decisions. Perspect Ecol Conserv **22**, 315–327. (10.1016/j.pecon.2024.11.004)

[29] Gardner TA *et al*. 2013 A social and ecological assessment of tropical land uses at multiple scales: the Sustainable Amazon Network. Phil. Trans. R. Soc. B **368**, 20120166. (10.1098/rstb.2012.0166) 23610172 PMC3638432

[30] Ter Steege H *et al*. 2023 Mapping density, diversity and species-richness of the Amazon tree flora. Commun. Biol. **6**, 1130. (10.1038/s42003-023-05514-6) 37938615 PMC10632362

[31] Hudson LN *et al*. 2017 The database of the PREDICTS (projecting responses of ecological diversity in changing terrestrial systems) project. Ecol. Evol. **7**, 145–188. (10.1002/ece3.2579) 28070282 PMC5215197

[32] Gibson J, Shokralla S, Porter TM, King I, van Konynenburg S, Janzen DH, Hallwachs W, Hajibabaei M. 2014 Simultaneous assessment of the macrobiome and microbiome in a bulk sample of tropical arthropods through DNA metasystematics. Proc. Natl Acad. Sci. USA **111**, 8007–8012. (10.1073/pnas.1406468111) 24808136 PMC4050544

[33] Ritter CD, Häggqvist S, Karlsson D, Sääksjärvi IE, Muasya AM, Nilsson RH, Antonelli A. 2019 Biodiversity assessments in the 21st century: the potential of insect traps to complement environmental samples for estimating eukaryotic and prokaryotic diversity using high-throughput DNA metabarcoding. Genome **62**, 147–159. (10.1139/gen-2018-0096) 30673361

[34] Magurran AE, Dornelas M, Moyes F, Henderson PA. 2019 Temporal β diversity—a macroecological perspective. Glob. Ecol. Biogeogr. **28**, 1949–1960. (10.1111/geb.13026)

[35] Stroud JT, Feeley KJ. 2017 Neglect of the tropics is widespread in ecology and evolution: a comment on clarke et al. Trends Ecol. Evol. (Amst.) **32**, 626–628. (10.1016/j.tree.2017.06.006) 28693756

[36] Clarke DA, York PH, Rasheed MA, Northfield TD. 2017 Does biodiversity-ecosystem function literature neglect tropical ecosystems? Trends Ecol. Evol. **32**, 320–323. (10.1016/j.tree.2017.02.012) 28279488

[37] Feeley K. 2015 Are we filling the data void? An assessment of the amount and extent of plant collection records and census data available for tropical South America. PLoS One **10**, e0125629. (10.1371/journal.pone.0125629) 25927831 PMC4416035

[38] Maestre FT, Eisenhauer N. 2019 Recommendations for establishing global collaborative networks in soil ecology. Soil Org. **91**, 73–85. (10.25674/so91iss3pp73) 31908680 PMC6944499

[39] Mammides C *et al*. 2016 Increasing geographic diversity in the international conservation literature: a stalled process? Biol. Conserv. **198**, 78–83. (10.1016/j.biocon.2016.03.030)

[40] Habel JC, Lens L, Eggermont H, Githiru M, Mulwa RK, Shauri HS, Lewinsohn TM, Weisser WW, Schmitt T. 2017 More topics from the tropics: additional thoughts to Mammides et al. Biodivers. Conserv. **26**, 237–241. (10.1007/s10531-016-1236-1)

[41] Baker S. 2023 North–south publishing data show stark inequities in global research. Nature **624**, S1–S1. (10.1038/d41586-023-03901-x)

[42] Huang J, Gates AJ, Sinatra R, Barabási AL. 2020 Historical comparison of gender inequality in scientific careers across countries and disciplines. Proc. Natl Acad. Sci. USA **117**, 4609–4616. (10.1073/pnas.1914221117) 32071248 PMC7060730

[43] Eisenhauer N, Hines J. 2021 Invertebrate biodiversity and conservation. Curr. Biol. **31**, R1214–R1218. (10.1016/j.cub.2021.06.058) 34637734

[44] IUCN. 2025 Summary statistics. See https://www.iucnredlist.org/resources/summary-statistics#Summary%20Tables (accessed 4 November 2025).

[45] Lapola DM *et al*. 2023 The drivers and impacts of Amazon forest degradation. Science **379**, eabp8622. (10.1126/science.abp8622) 36701452

[46] Barlow J *et al*. 2016 Anthropogenic disturbance in tropical forests can double biodiversity loss from deforestation. Nature New Biol. **535**, 144–147. (10.1038/nature18326) 27362236

[47] Warren R, Price J, Graham E, Forstenhaeusler N, VanDerWal J. 2018 The projected effect on insects, vertebrates, and plants of limiting global warming to 1.5°C rather than 2°C. Science **360**, 791–795. (10.1126/science.aar3646) 29773751

[48] Newbold T. 2018 Future effects of climate and land-use change on terrestrial vertebrate community diversity under different scenarios. Proc. R. Soc. B **285**, 20180792. (10.1098/rspb.2018.0792) PMC6030534 29925617

[49] Stegmann LF *et al*. 2024 Brazilian public funding for biodiversity research in the Amazon. Perspect Ecol Conserv **22**, 1–7. (10.1016/j.pecon.2024.01.003)

[50] Cámara-Leret R, Dennehy Z. 2019 Information gaps in indigenous and local knowledge for science-policy assessments. Nat. Sustain. **2**, 736–741. (10.1038/s41893-019-0324-0)

[51] Harris E. 2004 Building scientific capacity in developing countries. EMBO Rep. **5**, 7–11. (10.1038/sj.embor.7400058) 14710175 PMC1298969

[52] CNPq. 2025 Mapa de fomento em ciência,tecnologia & inovação—bolsas e projetos vigentes. See http://www.bi.cnpq.br/painel/mapa-fomento-cti.

[53] Shen H. 2013 Inequality quantified: Mind the gender gap. Nature **495**, 22–24. (10.1038/495022a) 23467149

[54] Bellotti E, Czerniawska D, Everett MG, Guadalupi L. 2022 Gender inequalities in research funding: Unequal network configurations, or unequal network returns? Soc. Networks **70**, 138–151. (10.1016/j.socnet.2021.12.007)

[55] Coelho MTP *et al*. 2023 The geography of climate and the global patterns of species diversity. Nature **622**, 537–544. (10.1038/s41586-023-06577-5) 37758942 PMC10584679

[56] Newbold T *et al*. 2019 Climate and land-use change homogenise terrestrial biodiversity, with consequences for ecosystem functioning and human well-being. Emerg. Top. Life Sci. **3**, 207–219. (10.1042/ETLS20180135) 33523149

[57] Luza AL *et al*. 2023 Beyond data labor: sowing synthesis science in the Global South. Perspect Ecol Conserv **21**, 265–270. (10.1016/j.pecon.2023.09.003)

[58] Spears BM *et al*. 2015 FORUM: effective management of ecological resilience—are we there yet? J. Appl. Ecol. **52**, 1311–1315. (10.1111/1365-2664.12497)

[59] Joly CA *et al*. 2019 Brazilian assessment on biodiversity and ecosystem services: summary for policy makers. Biota Neotrop **19**, 1–8. (10.1590/1676-0611-bn-2019-0865)

[60] Oliveira U *et al*. 2017 Biodiversity conservation gaps in the Brazilian protected areas. Sci. Rep **7**, 1–9. (10.1038/s41598-017-08707-2) 28831073 PMC5567310

[61] De Wever A, Schmidt-Kloiber A, Bremerich V, Freyhof J. 2018 Secondary data: taking advantage of existing data and improving data availability for supporting freshwater ecology research and biodiversity conservation. In Freshwater ecology and conservation (ed. J Hughes), pp. 306–320. Oxford, UK: Oxford University Press. (10.1093/oso/9780198766384.003.0014)

[62] Stephenson PJ, Stengel C. 2020 An inventory of biodiversity data sources for conservation monitoring. PLoS One **15**, e0242923. (10.1371/journal.pone.0242923) 33264320 PMC7710106

[63] ter Steege H. 1998 The use of forest inventory data for a national protected area strategy in Guyana. Biodivers Conserv **7**, 1457–1483. (10.1023/A:1008893920157)

[64] Zimmerman AS. 2008 New knowledge from old data. Sci. Technol. Human Values **33**, 631–652. (10.1177/0162243907306704)

[65] Chandler M *et al*. 2017 Contribution of citizen science towards international biodiversity monitoring. Biol. Conserv. **213**, 280–294. (10.1016/j.biocon.2016.09.004)

[66] Feng X *et al*. 2022 A review of the heterogeneous landscape of biodiversity databases: Opportunities and challenges for a synthesized biodiversity knowledge base. Glob. Ecol. Biogeogr. **31**, 1242–1260. (10.1111/geb.13497)

[67] Spironello W *et al*. 2023 Primates of Brazilian Amazonia: knowledge, research gaps, and conservation priorities. In Amazonian mammals (eds WR Spironello, AA Barnett, JW Lynch, PED Bobrowiec, SA Boyle), pp. 73–109. Cham, Switzerland: Springer. (10.1007/978-3-031-43071-8_4)

[68] Hortal J, de Bello F, Diniz-Filho JAF, Lewinsohn TM, Lobo JM, Ladle RJ. 2015 Seven shortfalls that beset large-scale knowledge of biodiversity. Annu. Rev. Ecol. Evol. Syst. **46**, 523–549. (10.1146/annurev-ecolsys-112414-054400)

[69] Stork NE. 2018 How many species of insects and other terrestrial arthropods are there on earth? Annu. Rev. Entomol. **63**, 31–45. (10.1146/annurev-ento-020117-043348) 28938083

[70] Wagner DL. 2020 Insect declines in the anthropocene. Annu. Rev. Entomol. **65**, 457–480. (10.1146/annurev-ento-011019-025151) 31610138

[71] Camacho‐Rozo CP, Urbina‐Cardona N. 2024 Major knowledge shortfalls for Colombian Amazonian anurans: Implications for conservation. Austral Ecol **49**, 1–21. (10.1111/aec.13564)

[72] ForestPlots.net *et al*. 2021 Taking the pulse of Earth’s tropical forests using networks of highly distributed plots. Biol. Conserv. **260**, 108849. (10.1016/j.biocon.2020.108849)

[73] de Lima RAF *et al*. 2015 How much do we know about the endangered Atlantic Forest? Reviewing nearly 70 years of information on tree community surveys. Biodivers. Conserv. **24**, 2135–2148. (10.1007/s10531-015-0953-1)

[74] Parr CL *et al*. 2017 GlobalAnts: a new database on the geography of ant traits (Hymenoptera: Formicidae). Insect Conserv. Diversity **10**, 5–20. (10.1111/icad.12211)

[75] Langer L, Erasmus Y, Tannous N, Stewart R. 2017 How stakeholder engagement has led us to reconsider definitions of rigour in systematic reviews. Environ. Evid **6**, 1–6. (10.1186/s13750-017-0098-7)31019679

[76] Gerlak AK *et al*. 2023 Stakeholder engagement in the co-production of knowledge for environmental decision-making. World Dev. **170**, 106336. (10.1016/j.worlddev.2023.106336)

[77] Visseren‐Hamakers IJ, Leroy P, Glasbergen P. 2012 Conservation partnerships and biodiversity governance: fulfilling governance functions through interaction. Sustainable Development **20**, 264–275. (10.1002/sd.482)

[78] Perez Arredondo AM, Yasobant S, Bruchhausen W, Bender K, Falkenberg T. 2021 Intersectoral collaboration shaping one health in the policy agenda: a comparative analysis of Ghana and India. One Health **13**, 100272. (10.1016/j.onehlt.2021.100272) 34136629 PMC8182263

[79] Norström AV *et al*. 2020 Principles for knowledge co-production in sustainability research. Nat. Sustain. **3**, 182–190. (10.1038/s41893-019-0448-2)

[80] Levis C *et al*. 2024 Contributions of human cultures to biodiversity and ecosystem conservation. Nat. Ecol. Evol. **8**, 866–879. (10.1038/s41559-024-02356-1) 38503867

[81] Vallet A *et al*. 2023 Knowledge coproduction to improve assessments of nature’s contributions to people. Conserv. Biol. **37**, e14182. (10.1111/cobi.14182) 37889094

[82] Räsänen A *et al*. 2024 Bridging the knowledge-action gap: a framework for co-producing actionable knowledge. Environ. Sci. Policy **162**, 103929. (10.1016/j.envsci.2024.103929)

[83] Howarth C, Lane M, Morse-Jones S, Brooks K, Viner D. 2022 The ‘co’ in co-production of climate action: challenging boundaries within and between science, policy and practice. Glob. Environ. Change **72**, 102445. (10.1016/j.gloenvcha.2021.102445)

[84] Nel JL *et al*. 2016 Knowledge co-production and boundary work to promote implementation of conservation plans. Conserv. Biol. **30**, 176–188. (10.1111/cobi.12560) 26041340

[85] Cooke SJ *et al*. 2021 Knowledge co-production: a pathway to effective fisheries management, conservation, and governance. Fisheries **46**, 89–97. (10.1002/fsh.10512)

[86] Adelle C, Pereira L, Görgens T, Losch B. 2020 Making sense together: the role of scientists in the coproduction of knowledge for policy making. Sci. Public Policy **47**, 56–66. (10.1093/scipol/scz046)

[87] van der Hel S. 2016 New science for global sustainability? The institutionalisation of knowledge co-production in future earth. Environ. Sci. Policy **61**, 165–175. (10.1016/j.envsci.2016.03.012)

[88] Martínez‐Harms MJ, Estévez RA, Álvarez‐Miranda E. 2024 Conservation triage in action: planning, governance and knowledge co‐production for biodiversity protection. Journal of Applied Ecology **61**, 2328–2334. (10.1111/1365-2664.14763)

[89] Schick A, Sandig C, Krause A, Hobson PR, Porembski S, Ibisch PL. 2018 People-centered and ecosystem-based knowledge co-production to promote proactive biodiversity conservation and sustainable development in namibia. Environ. Manage. **62**, 858–876. (10.1007/s00267-018-1093-7) 30120499

[90] Feitosa RA. 2021 New public policy for teacher training in Brazil: Vincent van Gogh as an inspiration for the action of the pedagogical residency program? Policy Futures in Education **19**, 28–43. (10.1177/1478210320940129)

[91] Wang K, Lee CS, Marinkovich MP, Chang HY, Oro AE, Khavari PA. 2016 Factors that may promote an effective local research environment. J. Invest. Dermatol. **136**, 1529–1531. (10.1016/j.jid.2016.04.015) 27450496 PMC5873952

[92] Seguin B, State L, Singer PA, Daar AS. 2006 Scientific diasporas as an option for brain drain: re-circulating knowledge for development. Int. J. Biotechnol **8**, 78–90. (10.1504/IJBT.2006.008965)

[93] Docquier F, Lohest O, Marfouk A. 2007 Brain drain in developing countries. World Bank Econ. Rev. **21**, 193–218. (10.1093/wber/lhm008)

[94] Larivière V, Gingras Y, Sugimoto CR, Tsou A. 2015 Team size matters: collaboration and scientific impact since 1900. J. Assoc. Inf. Sci. Technol. **66**, 1323–1332. (10.1002/asi.23266)

[95] Klug M, Bagrow JP. 2016 Understanding the group dynamics and success of teams. R. Soc. Open Sci. **3**, 160007. (10.1098/rsos.160007) 27152217 PMC4852640

[96] Zhu N, Liu C, Yang Z. 2021 Team size, research variety, and research performance: do coauthors’ coauthors matter? J. Informetr. **15**, 101205. (10.1016/j.joi.2021.101205)

[97] Plex Sulá AI *et al*. 2024 What traits of collaboration networks are associated with project success? The case of two CGIAR agricultural research programs for development. Agric. Syst. **219**, 104013. (10.1016/j.agsy.2024.104013)

[98] Wuchty S, Jones BF, Uzzi B. 2007 The increasing dominance of teams in production of knowledge. Science **316**, 1036–1039. (10.1126/science.1136099) 17431139

[99] Jenkins LD, Maxwell SM, Fisher E. 2012 Increasing conservation impact and policy relevance of research through embedded experiences. Conserv. Biol. **26**, 740–742. (10.1111/j.1523-1739.2012.01878.x) 22809354

[100] Yang L. 2021 How does the local knowledge of scientists influence their impact on governance performance? Sci. Public Policy **48**, 334–352. (10.1093/scipol/scab002)

[101] Colares. 2025 Brazilian National Institutes of Science and Technology repository. Zenodo. (10.5281/zenodo.17391954)

[102] Oliveira de Resende BO *et al*. 2025 Supplementary material from: Collaborative research networks as a strategy to synthesize knowledge of Amazonian biodiversity. Figshare. (10.6084/m9.figshare.c.8108725) PMC12646749 41290183

